# Do Combinations of Behavior Change Techniques That Occur Frequently in Interventions Reflect Underlying Theory?

**DOI:** 10.1093/abm/kaaa078

**Published:** 2020-09-22

**Authors:** Lauren Connell Bohlen, Susan Michie, Marijn de Bruin, Alexander J Rothman, Michael P Kelly, Hilary N K Groarke, Rachel N Carey, Joanna Hale, Marie Johnston

**Affiliations:** 1 Department of Clinical, Educational and Health Psychology, University College London, Torrington Place, London, UK; 2 Department of Behavioral and Social Sciences, Brown University School of Public Health, Providence, RI, USA; 3 Aberdeen Health Psychology Group, Institute of Applied Health Sciences, College of Life Sciences and Medicine, University of Aberdeen, Aberdeen, UK; 4 Radboud University Medical Center, Radboud Institute of Health Sciences, Nijmegen, Netherlands; 5 Department of Psychology, University of Minnesota, Minneapolis, MN, USA; 6 Institute of Public Health, University of Cambridge, Cambridge, UK

**Keywords:** Behavior change theory, Multicomponent intervention, Intervention design, Intervention evaluation

## Abstract

**Background:**

Behavioral interventions typically include multiple behavior change techniques (BCTs). The theory informing the selection of BCTs for an intervention may be stated explicitly or remain unreported, thus impeding the identification of links between theory and behavior change outcomes.

**Purpose:**

This study aimed to identify groups of BCTs commonly occurring together in behavior change interventions and examine whether behavior change theories underlying these groups could be identified.

**Methods:**

The study involved three phases: (a) a factor analysis to identify groups of co-occurring BCTs from 277 behavior change intervention reports; (b) examining expert consensus (*n* = 25) about links between BCT groups and behavioral theories; (c) a comparison of the expert-linked theories with theories explicitly mentioned by authors of the 277 intervention reports.

**Results:**

Five groups of co-occurring BCTs (range: 3–13 BCTs per group) were identified through factor analysis. Experts agreed on five links (≥80% of experts), comprising three BCT groups and five behavior change theories. Four of the five BCT group–theory links agreed by experts were also stated by study authors in intervention reports using similar groups of BCTs.

**Conclusions:**

It is possible to identify groups of BCTs frequently used together in interventions. Experts made shared inferences about behavior change theory underlying these BCT groups, suggesting that it may be possible to propose a theoretical basis for interventions where authors do not explicitly put forward a theory. These results advance our understanding of theory use in multicomponent interventions and build the evidence base for further understanding theory-based intervention development and evaluation.

## Teaser

The theory underlying behavior change interventions is important for understanding how and why they work, but may not be made explicit in published reports. This study found that experts in behavior change can agree on some of these theories by looking at the combinations of behaviour change techniques used in these interventions.

Developing an efficacious behavior change intervention is a complex process. One approach is to select a theory or generate a hypothesis about the mechanisms of action that need to be targeted to promote behavior change and choose intervention techniques that can elicit changes in those targets. Theories help to make sense of the complexity of behavior and behavior change by providing varying degrees of specification of why and how behavior change occurs, under what circumstances, and for whom. Theories provide some guidance regarding potential targets for intervention (causal determinants of behavior), which can inform which intervention techniques to use. The early stage of moving from behavior change theory to behavior change techniques (BCTs) may be described explicitly or may be implicit and not fully reported. Despite the recognized importance of the systematic application of theory to the design of interventions [1–6], there is variability in the reported use of theory to develop interventions. A clear, theory-based rationale for an intervention provides a way to understand how interventions have their effects and permits testing and refining the theory itself [1]. It follows that, if the theoretical rationale underlying intervention development is not explicitly reported, evidence from interventions based on the theory cannot be synthesized as evidence relating to the theory, thus impeding progress in both theory and intervention development. However, it is plausible that even when theories are not explicitly reported, intervention techniques are not selected at random, and there are identifiable systematic patterns of combinations of intervention techniques, and those combinations may map onto theory.

Most behavior change interventions use combinations of multiple BCTs, that is, the active ingredients within an intervention that lead to behavior change. The use of multiple BCTs within an intervention does not in itself necessarily increase intervention effectiveness: however, interventions that use a combination of BCTs aligned with a behavior change theory have been associated with increased intervention effectiveness [7–9]. Thus, building a map of how BCTs might work together synergistically based on theory either from an explicit understanding (i.e., the theory identifies specific BCTs) or an implicit understanding (i.e., BCTs are not explicitly identified, but theory identifies a set of intervention targets) is likely to advance the science of behavior change by improving our ability to explore links between theory and intervention effectiveness.

In some cases, a theory gives no guide to the specific techniques that should be used to change behavior. For example, in discussing the development of behavior change interventions based on the theory of planned behavior, Ajzen writes: “Once it has been decided which beliefs the intervention will attempt to change, an effective intervention method must be developed. This is where the investigator’s experience and creativity come into play. The Theory of Planned Behavior can provide general guidelines ……but it does not tell us what kind of intervention will be most effective.” [10]. Some theories do address, in part, how to change behavior. For example, theories of risk perception specify that behavior change will be achieved when fear arousal is combined with techniques to increase perceived self-efficacy and response efficacy, although the techniques needed to do so are not specified. Interventions that use fear arousal alone as an intervention technique have generally been ineffective (or, in some cases, counter-productive) compared to interventions that are more theoretically aligned and use fear-arousal techniques coupled with techniques to change self-efficacy and response efficacy [11].

In some instances, an intervention’s underlying theory can retrospectively be deduced based on the match between the group of BCTs used and those specified in a theory. In cases where a theory does not specify BCTs, one can work in reverse to identify an underlying theory when the mechanisms of action in the intervention can be mapped to theory. Using this methodology, Gardner et al. were able to make sense of mixed evidence on the effectiveness of “audit & feedback” in changing clinical behaviors; interventions that used a combination of techniques targeting the mechanisms specified by control theory (behavioral targets, action plans, and feedback) were most likely to be effective. Given that not all theories specify intervention techniques, intervention designers select multiple BCTs to target change in theoretical processes. Frequently co-occurring groups of BCTs may reflect a shared implicit understanding of a theoretically based synergistic or additive relationship between those intervention techniques based on behavior change processes described within the theory.

In this study, we investigate whether there are frequently occurring combinations of BCTs used in behavior change interventions and whether these can be linked to behavior change theories. First, we examined the extent to which groups of BCTs occur together in published reports of interventions (RQ1). Second, we examined whether behavior change experts could agree about the links between behavior change theories and the identified groups of BCTs (RQ2). Finally, where authors *explicitly reported* an underlying theory for an intervention, we examined whether the theories identified by experts matched the theories explicitly stated by the authors of interventions incorporating the majority of BCTs in any of the identified groups (RQ3).

## Methods

### Study Design

The study used evidence from a data set of 277 behavior change intervention reports published between 1982 and 2016, covering 10 different behavioral domains that were generated in a previous literature synthesis study [12] in which each intervention was systematically coded for BCTs using the Behavior Change Techniques Taxonomy v1 (BCTTv1). Details about inclusion criteria are included in the original manuscript [12]; papers were included if the behavior change intervention was described and if at least one BCT was identified and described by the authors as linked to at least one mechanism of action. This study consisted of three phases. First, a factor analysis of the BCTs identified in interventions in the literature synthesis study examined the extent to which BCTs tend to co-occur across interventions (RQ1). Second, a modified nominal group technique established expert consensus about links between each of the BCT groups and theories of behavior change (RQ2). Third, the data set of intervention reports was reexamined to identify interventions that used a majority of the BCTs from one of the BCT groups and where authors explicitly based their selection of BCTs on a theory of behavior change (RQ3). Using this information, the similarity of the BCT group–theory links identified through expert consensus was compared with those specified in the literature by the intervention authors.

### Phase 1: Identify Groups of BCTs That Frequently Co-occur in Published Intervention Reports

#### Procedure

BCTs extracted from peer-reviewed intervention reports (*n* = 277) [12] were used to identify groups of BCTs that appeared frequently together across interventions. To identify BCT groups, exploratory factor analysis was used to account for the extent to which BCTs are correlated across interventions. This method permitted data-driven identification of co-occurring groups of BCTs without imposing any hypothesized structure on the observed variables [13]. The factors or latent variables identified might reflect theorizing of a synergistic or additive relationship.

#### Analyses

The data used to identify BCT groups were generated from the presence or absence of BCTs in reports of 277 behavior change interventions. An exploratory factor analysis was conducted to identify groups of two or more BCTs that co-occurred across interventions, considering the binary nature of the data. Prior research suggests that, when estimating factors with an average loading between .4 and .70, a sample size of approximately 200 is needed and that, as the average loading per factor decreases, larger sample sizes are needed [14]. To produce a stable factor solution with reliable factors, we trimmed the minimum number of BCTs from the analysis. BCTs that occurred in fewer than 5% of all interventions were excluded (*n* = 29). Lower frequency BCTs would by nature have lower factor loadings, and increasing the sample size (i.e., number of papers) would not necessarily increase the occurrence of low-frequency BCTs (i.e., [15]). This resulted in a factor analysis of 48 different BCTs present within the interventions. Exploratory factor analyses were conducted in Mplus v8, which allows for factor analysis of categorical and/or binary variables using the maximum likelihood estimator [16]. All data entered into the analysis were binary (BCT presence vs. absence), and an oblique rotation (i.e., geomin rotation [16]) was used. An oblique rotation was chosen in order to allow the factors (the BCT groups) to be correlated; this permitted the assumption that certain groups of BCTs that co-occur across interventions might also co-occur together within interventions. Similarly, the geomin rotation was used to permit the assumption that BCTs might load highly on more than one factor.

##### Determining the number of factors retained

Three sources of information determined the number of factors retained. First, factors with eigenvalues greater than one were retained [13] and scree plots were examined to further specify the number of factors (Supplementary File 1). Second, to determine whether the number of factors chosen fits the structure of the data well, multiple indices of model fit were examined. Multiple indices of model fit provide different information for determining model fit and a more conservative and reliable estimation of model fit [17, 18]. Acceptable model fit to determine the appropriate number of factors was determined using the following criteria: root mean square error approximation <.05, comparative fit index >.90, and the *p*-value for chi-square >0.05 [19]. The best-fitting factor solutions were then further examined to exclude those factor solutions containing a larger number of BCTs that loaded onto multiple factors. Due to the nature of the data analyzed, there were no missing data.

##### Identifying BCT factor group membership

After identifying the factor solution with the least number of cross-loading BCTs, the factor loadings were used to determine which BCTs co-occurred within each of the factors retained. Since there were a large number of variables analyzed and only a sample of 277 interventions, we used a conservative criterion established by prior research to determine which BCTs met the criterion for factor membership; only those BCTs that had a factor loading ≥.45 on a given factor were retained [20, 21]. In the instance a BCT loaded on more than one factor with a factor loading >.45, the BCT was retained on the factor for which it had the highest loading, with the assumption that the higher the factor loading, the more frequently the BCT co-occurred with the other BCTs in the group.

### Phase 2: Expert Consensus to Identify Links Between BCT Groups and Theories

#### Participants

Participants were 25 experts who designed, evaluated, and/or synthesized evidence about theory-based behavior change interventions. Experts were recruited from a pool of 100 behavior change experts who participated in a previous consensus study linking BCTs to theoretical mechanisms of action (eligibility criteria described elsewhere [22]) and who demonstrated advanced knowledge of behavioral theories and BCTs [22, 23]. Experts were selected according to the following criteria: (a) self-reported “extensive” publication history of interventions that specify behavior change theory and (b) active participation rate in the previous consensus study, defined as commenting on at least half of the links available for discussion (i.e., greater than 11 comments in the previous exercise). Fifty-six percent of experts were from the UK, 24% were from other European countries, 16% were from the USA or Canada, and 4% were from Australia; 68% of experts had a psychology background. Expertise was evaluated using self-reported ratings of expertise in behavior change interventions, BCTs, and behavior change theories (for more information, see [22, 23]). At the time of participation in this study, 92% of experts (*n* = 23) worked in the university sector and 96% (*n* = 24) had doctoral-level training. Expert panels of 20 or more members have been found to be effective in establishing consensus and have shown stability in agreement across a similar number of rounds in previous consensus studies [24].

#### Materials

For each round of the consensus exercise, experts were provided with the retained BCTs for each factor identified in Phase 1, presented as “BCT groups” with the factor loading for each BCT, along with the definition of each BCT in the group, based on BCTTv1 [15] (Supplementary File 2). Experts received an online copy of the “ABC of Behavior Change Theories” book [25], which describes 83 theories used in behavior change interventions to enable them to select an appropriate theory for each group. Participants were asked to consider each group and propose a theory (or theories), if any, on which this combination of BCTs might be based. Experts also had the option to not list any theories for a group of BCTs. To ensure consistency across experts, and with the aim of developing a shared understanding of BCT group–theory links, experts were asked to base their answers on the definitions of BCTs rather than the BCT label. Supplementary File 3 presents full task guidelines for completing each round.

#### Procedure

All procedures involving human subjects were approved by the university ethics committee at University College London. A modified consensus development method, drawing on Nominal Group Technique [26, 27] was adopted to develop expert consensus about BCT group–theory links. This took place online and involved four rounds: (a) an open response task round to generate links between BCT groups and theories, (b) an initial rating round to gauge consensus around each BCT group–theory link, (c) a discussion round to address links lacking consensus, and (d) a final rating round to establish final levels of consensus for each link. Expert ratings in Rounds 1, 2, and 4 were made using Qualtrics survey software [28], and the discussion in Round 3 was hosted via the online forum Loomio [29]. Information about informed consent was emailed to participants prior to the start of Round 1, and consent was obtained via Qualtrics.

##### Round 1

Experts participated in an open response task to list all possible theories underlying each BCT group. Experts were instructed by email to draw from their own knowledge and expertise and/or the 83 theories from the “ABC of Behavior Change Theories” book [25].

##### Round 2

Experts were presented with the same BCT groups as in Round 1 along with all theories listed for each group by more than one expert in Round 1. To reduce participant burden and maximize the possibility for consensus, theories needed to be mentioned by more than one expert in Round 1. The order of the groups and theories was randomized. Each BCT group appeared individually on the screen and experts rated how confident they were the group should be linked to a given theory on a three-point scale (Very confident, Uncertain/Don’t know, and Not at all confident). BCT group–theory links with confirmed consensus in Round 2 (criterion: ≥80% experts provided “Very confident” or “Not at all confident” responses) were not subsequently presented for discussion or expert ratings in Rounds 3 and 4.

##### Round 3

Experts contributed to an anonymous, asynchronous discussion to exchange views about BCT group–theory links that lacked consensus following Round 2. Experts were prompted to discuss links with high uncertainty (i.e., high percentage of “Uncertain/Don’t Know” responses) and links with high disagreement (i.e., nearly equivalent proportions of experts rating “Very confident” and “Not at all confident”). This allowed experts to discuss uncertainties and disagreements to help guide the final ratings in Round 4. Before the task, experts were provided with data from Round 2, including a summary of all experts’ responses alongside their own. These data were also presented during the task, and experts were advised to look at the data and reflect before contributing to the discussion. Experts were sent a link to the discussion forum via email where they were prompted to register a Loomio account and log in using an assigned Expert ID. The forum contained separate discussion threads for each BCT group–theory link to be discussed. There was also a space for experts to contribute thoughts about other BCT group–theory links and/or about the task more generally. Experts were advised to focus on the ratings of BCT group–theory links for which they remained uncertain or where they disagreed with other experts. A moderator from the research team periodically summarized the discussion and raised issues for further consideration.

##### Round 4

Experts provided final ratings of their certainty about the BCT group–theory links. Round 4 ratings could be the same as the initial ratings provided in Round 2. Experts were provided with Round 2 ratings for each BCT group–theory link and a hyperlink to the Loomio discussion page if discussed in Round 3. Links that reached consensus in Round 2 were not included. As in Round 2, experts were presented with the five BCT groups individually and asked to report their confidence in the link to a particular theory on a three-point scale (Very confident, Uncertain/Don’t know, and Not at all confident). These final ratings were used for the present analyses.

Experts were also asked to indicate up to three theories they were most confident were linked to a group of BCTs. To examine the extent to which the entirety of a BCT group might link to a given theory, for each of the three theories selected, experts could add and/or remove BCTs from each respective group. Experts were informed that they could base their judgments on the strength of links between the BCTs (i.e., factor loadings) and the constructs of a theory.

##### Data analysis

Descriptive statistics for consensus exercise rounds were generated (in *MS Excel*). For Round 1, we examined the number of unique theories listed by experts, the number of theories linked to each BCT group, and the number of theories linked to more than one BCT group. For Round 3, we examined the total number of comments per Loomio discussion thread and the total number of comments per expert. Round 2 and Round 4 data were used to identify which BCT groups are most frequently linked to theories by experts (RQ2). The criterion for consensus was ≥80% of experts rating they were “Very confident” or “Not at all confident” that a BCT group and a theory were linked. Data from Round 4 were used to identify which theories experts were the most confident were linked to a specific BCT Group. Separate analyses were conducted to determine the frequency with which experts rated whether a BCT should be added or removed from a BCT Group.

### Phase 3: Comparison of Expert-Agreed BCT Group–Theory Links With Published Reports

#### Procedure

To compare theories generated in the consensus exercise with those in the original intervention reports, the data set of 277 published intervention reports was used [12]. The data set included a list of all BCTs used in each intervention article coded using BCTTv1 [15].

Intervention reports were extracted, which used more than half of the BCTs within any group. The extracted interventions were then screened to identify whether and how explicitly behavior change theories guided the development of an intervention that used a majority of the BCTs from a given BCT group. Intervention reports were only included in this analysis where the authors stated that the development of the intervention was grounded in a theory.

#### Data analysis

One researcher coded the selected intervention reports according to how explicitly theory was used in developing the intervention (0 = no theory mentioned; 1 = theory mentioned but not specified as underlying interventions; and 2 = theory-guided intervention) and the name of each specific theory mentioned in the report. This information was tabulated (Table 3) and used to calculate the total number of intervention reports where a BCT group was referenced (at least in part) and theory of behavior change specified. A second researcher randomly checked 20% of the final table for accuracy, and discrepancies were resolved through discussion.

To determine if the BCT group–theory links identified by experts are similar to the BCT group–theory links that appeared in intervention reports (RQ3), we compared the Phase 2 and 3 results in a frequency table. This allowed us to identify the evidence of convergence between the BCT group–theory links generated by the two sources of evidence.

## Results

### Phase 1: Identify Groups of BCTs That Frequently Co-occur in Published Intervention Reports

#### The factor solution

Examination of the eigenvalues and scree plot from an initial exploratory factor analysis detected a factor solution between one and eight factors. Although 16 factors had an eigenvalue greater than 1, the change in eigenvalues became increasingly small and consistent after eight factors. Factor solutions with five, six, seven, and eight factors had a satisfactory model fit across multiple fit indices (Table 1). The five-factor solution was judged to have the most acceptable model fit, with all prespecified criteria for fit met, and it offered the most parsimonious solution. Overall the BCT groups were not very highly correlated, despite a significant interfactor correlation between BCT Group 2 and two other BCT groups (see Table 2).

**Table 1.** Model fit indices for exploratory factor analysis solutions for between one and eight factors (behavior change technique [BCT] groups)

	RMSEA	CFI	Chi-square (*df*), *p*-value
Target values for acceptable model fit	<.05	>.90	*p* > .05
1 Factor	.034	.746	1,219.28 (1,080), .001
2 Factors	.030	.802	1,141.23 (1,033), .01
3 Factors	.027	.845	1,072.09 (987), .03
4 Factors	.024	.891	1,001.81 (942), .09
5 Factors	.019	.932	935.27 (898), .19
6 Factors	.015	.960	876.23 (855), .30
7 Factors	.013	.973	827.62 (813), .35
8 Factors	.005	.996	774.32 (772), .47

*CFI* comparative fit index; *df* degrees of freedom; *RMSEA* root mean square error approximation.

**Table 2.** Factor correlation matrix for the five-factor solution

	1	2	3	4
BCT Group 1	1.00			
BCT Group 2	.191*	1.00		
BCT Group 3	.076	.141	1.00	
BCT Group 4	.122	.192	.020	1.00
BCT Group 5	.206	.274*	.062	.112

All correlation coefficients (*r*) indicate the correlation between the two designated BCT groups,*p < .05.

#### Co-occurring BCTs by factor

Assignment of BCTs to factors using the criterion factor loadings >.45 resulted in 29 (of the 48) BCTs assigned to five different factors. BCTs with higher factor loadings are most descriptive of the BCT group. The highest factor loadings in a group indicate which BCTs co-occur most often across interventions. One BCT (12.3 Avoidance/reducing exposure to cues for the behavior) had a factor loading greater than .5 for more than one factor (Groups 3 and 5). This BCT was selected to load on BCT Group 3 due to a higher factor loading. A table of all factor loadings greater than .45 for all five factors is presented in rank order in Table 4.

Each BCT group contained between 3 and 13 BCTs. Several of the BCTs contained within a BCT group also belonged to clusters identified in BCTTv1. For example, in BCT Group 1, 7 of the 13 BCTs are part of the BCTTv1 cluster “Goals and Planning” [15]. In BCT Group 2, two of the three BCTs are in BCTTv1 cluster “Feedback and Monitoring.” BCT Group 3 had seven BCTs, three of which belong to the BCTTv1 cluster “Natural Consequences” and two of which belong to “Antecedents.” None of the BCTs in Groups 4 or 5 mapped onto BCTTv1 clusters.

### Phase 2: Expert Consensus to Identify Links Between BCT Groups and Theories

#### Round 1

All experts invited to participate agreed (*n* = 25) and participated in the Round 1 open response task where they listed all possible theories that could underlie each of the five BCT groups. During this task, experts listed a total of 81 unique theories across the five BCT groups; five of the theories listed did not come from the textbook provided for the task.

Seventy-five unique theories were linked to a BCT group by more than one expert and thus carried forward to Round 2. Experts listed between 1 and 68 theories per BCT group. A total of 36 theories were listed by experts as potentially underlying BCT Group 1; 45 theories were listed for BCT Group 2; 68 theories were listed for BCT Group 3; 25 for BCT Group 4; and 20 for BCT Group 5. Eight theories were proposed as potentially underlying all five BCT groups: the COM-B Model, Health Action Process Approach, Integrated Theory of Health Behavior Change, Relapse Prevention Model, Self-Determination Theory, Social Cognitive Theory, Self-Efficacy Theory, and Theory of Planned Behavior. Ten theories were proposed to be linked to four BCT groups. All theories suggested for each BCT group are in Supplementary File 4.

#### Round 2

All experts (*n =* 25) participated in Round 2 and provided initial ratings about their confidence in the link between theories linked to a BCT group by more than one expert (a total of 194 BCT group–theory pairings). There was consensus for eight BCT group–theory pairings: for four pairings, at least 80% of experts were “very confident” the BCT group and the theory were linked (2% of all pairings). BCT Group 1 linked with both “Health Action Process Approach” and “Self-Regulation Theory.” BCT Group 4 linked with “Self Efficacy Theory” and “Social Cognitive Theory.” For four other pairings, at least 80% of experts were “not at all confident” they were linked (2% of all pairings). At least 50% of experts were “very confident” in a further 31 links (16% of all pairings), “uncertain” about 10 links (5.72% of all pairings), and “not at all confident” about 57 links (29% of all pairings). For 23 BCT group–theory links, there was high disagreement (*n* = 11) and/or uncertainty (*n* = 12) among experts, defined as nearly equal numbers of experts rating “very confident” and “not at all confident” and more than 50% of experts indicating they were “uncertain.”

#### Round 3

All experts *(n* = 25) participated in Round 3, discussing the 23 links with high disagreement or uncertainty from Round 2. Each of these BCT group–theory links had separate discussion threads in addition to a discussion thread for experts to discuss the consensus exercise in general. The total number of comments per discussion thread ranged from 7 to 22 (*M* = 12.29, standard deviation [*SD*] = 4.06) and the total number of comments per expert ranged from 1 to 25 (*M* = 11.6, *SD* = 7.08).

Experts found the rating task difficult for various reasons, including uncertainty about how many BCTs in the BCT group needed to link to the theory:

…a little confused about the task, particularly in terms of whether all the different techniques had to relate to the theory in question, or whether just one of them might. Because in practice, when developing an intervention, people often draw on several different theories.

Some experts noted how a lack of familiarity with some theories impacted their confidence ratings:

I was not aware of the Action theory model of consumption and I am not familiar with consumer behavior. Mostly these aspects made me mark “not at all confident.”

I’m not very familiar with PRIME theory so not sure I could ever be “very confident” about making a judgement about it!

Experts believed more familiar theories appeared to receive higher confidence ratings, regardless of their suitability to a BCT group:

When I saw the percentages of agreement by other experts in the final round I got the feeling that those theories that are well known to health psychologists received higher confidence ratings than rather unknown theories - no matter whether the BCT groups really fitted.

Several experts indicated difficulty with the task due to the number of theories included:

As there were so many theories involved I guess that none of the invited experts were experts for each of those theories. […] I had to look up most of the theories, because the exercise made me more and more confused about what I thought I knew about the theories I work most with as there was just too much information to process.

Despite difficulty with the task, experts found the discussion exercise helpful:

The discussion on this one has been very persuasive. I originally put uncertain for many of the reasons other have highlighted above. From reading the justifications given above, I am happy to change to “not confident.”

#### Round 4

All but one expert (*n* = 24, 96%) participated in Round 4, providing final confidence ratings about the pairings of BCT groups and theories for which consensus had not been achieved in Round 2 (a total of 186 pairings). Consensus was reached that over 80% of experts were “very confident” about one link (0.54%) between BCT Group 3 and the Theory of Planned Behavior and “not at all confident” about 13 links (7%). Expert consensus did not emerge for any of the 23 links for which there was high disagreement and/or uncertainty following Round 2.

Of the 81 theories generated in Round 1, 10 experts (42% of experts) reported that the Theory of Planned Behavior was the theory they were most confident about being linked to BCT Group 3. Despite 42% of experts linking BCT Group 3 to the Theory of Planned Behavior, 6 of the 10 experts who reported that they were “most confident” in the link indicated that they would not maintain all the seven BCTs in the group given a link to the theory. Three experts (12.5%) chose to remove BCT 13.2 *Framing/reframing* from the BCT Group, and three removed BCT 12.3 *Avoidance/reducing exposure to cues for the behavior*.

Although consensus was not reached for any theory with BCT Groups 2 and 5, 13 experts (54%) rated Control Theory, 12 experts (50%) rated Feedback Intervention Theory, and 10 experts (42%) rated Self-Regulation Theory in their top three most confident theories to be linked to BCT Group 2. Similarly, 12 experts (50%) rated Operant Learning Theory and 8 (33%) rated Self-Efficacy Theory in their top three most confident theories for BCT Group 5.

#### Consensus exercise outcome

At the conclusion of the exercise, consensus was reached that experts were “very confident” in a total of five BCT group–theory links (four from Round 2 plus one from Round 4; see Table 4) and “not at all confident” in a total of 17 links (four from Round 2 plus 13 from Round 4; Supplementary File 5). Some BCT groups were agreed to be linked to more than one theory, but no theory was agreed to be linked to more than one BCT group.

### Phase 3: Comparison of Expert-Agreed BCT Group–Theory Links With Published Reports

A total of 177 (64%) of 277 intervention reports contained at least half of the BCTs from one BCT group, including 48 (17%) that reflected more than one BCT group. Table 3 shows the number of intervention reports for each BCT group, broken down by the exact number of BCTs included. Of the 177 intervention reports, 168 explicitly stated that intervention development was grounded in a theory. Table 3 also shows the number of theory-guided interventions by BCT group. Only these 168 intervention reports were selected to identify BCT group–theory links as reported by authors. Table 4 compares the evidence for links between BCT groups and theory based on the theories identified in the consensus exercise and the theories explicitly identified in the intervention reports where the BCTs used reflected a majority of BCTs from one of the five co-occurring groups. For Group 1, experts linked two theories, “Health Action Process Approach” and “Self Regulation Theory.” The comparable links in the literature were not well supported, “Health Action Process Approach” was explicitly stated as the underlying theory in 3 of 25 intervention reports, and “Self-Regulation Theory” was not stated in any of the reports. Experts did not identify any links to theory for Group 2 yet, in the literature, “Social Cognitive Theory” was referenced 17 of 82 times theories were reported in interventions that used a majority of the BCTs in Group 2. In Group 3, which experts linked to “Theory of Planned Behavior,” of interventions using a majority of the BCTs from this BCT Group, three authors explicitly reported the same theory. In Group 4, the theories identified by experts, “Social Cognitive Theory” and “Self-Efficacy Theory,” were referenced 46 (40 and 6, respectively) of the 152 times theories were mentioned in interventions using a similar set of BCTs. For Group 5, experts did not identify links to a theory, but authors most commonly referred to “Social Cognitive Theory” and “Self-Efficacy Theory” (7 out of 15 reports) in intervention reports that used a majority of the same BCTs as those found in BCT Group 5.

**Table 3.** Frequency of the number of intervention reports reflecting a BCT group, broken down by the number of BCTs included

	BCT Group 1				BCT Group 2		BCT Group 3			BCT Group 4		BCT Group 5	
BCTs in BCT group (*n)*	13				3		7			3		3	
Intervention reports (*n)*	19				73		12			122		13	
Theory-guided interventions (*n)*	14				40		9			94		11	
BCTs from BCT group used in intervention (*n*)	7	8	9	10	2	3	4	5	7	2	3	2	3
Intervention reports with [*N*] BCTs from BCT group (*n*)	6	9	2	2	54	19	7	3	2	66	56	11	2
Included BCTs	1.5 Review behavior goals 1.4 Action planning 15.3 Focus on past success 1.3 Goal setting (behavior) 15.4 Self-talk 3.3 Social support (emotional) 1.2 Problem solving 2.3 Self-monitoring of behavior 8.7 Graded tasks 1.3 Goal setting (outcome) 1.5 Discrepancy between current behavior and goal 2.4 Self-monitoring of outcomes of behavior 1.8 Behavioral contract				2.7 Feedback on outcomes of behavior		5.2 Salience of consequences			8.1 Behavioral practice/rehearsal		12.6 Body changes	
					2.2 Feedback on behavior		12.3 Avoidance/reducing exposure to cues for the behavior						
					6.2 Social comparison					6.1 Demonstration of the behavior			
												10.9 Self-reward	
													
							6.3 Information about other’s approval			4.1 Instruction on how to perform the behavior		11.2 Reduce negative emotions	
							12.2 Restructuring the social environment						
													
							5.3 Information about social and environmental consequences						
							5.1 Information about health consequences						
							13.2 Framing/reframing						
													
													

*BCT* behavior change technique; *N* = number of BCTs from BCT group used in intervention; *n =* frequency.

**Table 4.** Frequency table comparing Phase 2 and Phase 3 behavior change technique (BCT) Group—theory links

BCTs in BCT Group	Frequency in intervention reports	Factor loading	Theories identified by expert consensus	Experts “Very Confident” in the link	Theories identified in published intervention reports	Intervention reports with link
	*n*			%		*n*
BCT Group 1						
1.5 Review behavior goals	36	.823	**Health action process approach**	84	Social cognitive theory	5
1.4 Action planning	115	.783		80	Self-determination theory	4
15.3 Focus on past success	32	.729	Self-regulation theory		Theory of planned behavior (TPB)	3
1.3 Goal setting (behavior)	145	.717			**Health action process approach (HAPA)**	3
15.4 Self-talk	16	.712			Transtheoretical Model (TTM)	1
3.3 Social support (emotional)	14	.705			Information-Motivation-Behavior (IMB) skills model	1
1.2 Problem solving	145	.624			Control theory	1
2.3 Self-monitoring of behavior	95	.613			Chronic disease self-management model	1
8.7 Graded tasks	47	.570				
1.3 Goal setting (outcome)	20	.560			I-change	1
1.6 Discrepancy between current behavior and goal	17	.523			Motivational interviewing	1
					Relative deprivation	1
2.4 Self-monitoring of outcomes of behavior	21	.503			Limited resources	1
1.8 Behavioral contract	30	.489			Narrative transportation theories	1
					Implementation intentions	1
BCT Group 2						
2.7 Feedback on outcomes of behavior	28	.807			Social cognitive theory	17
2.2 Feedback on behavior	114	.740			TPB	9
6.2 Social comparison	102	.626			Self-efficacy theory	5
					Self-determination	4
					TTM	3
					IMB skills model	3
					Operant learning theory	3
					Precaution adaptation process model	3
					I-change	3
					HAPA	2
					Health belief model	2
					Control theory	2
					Social comparison	2
					Theory of Reasoned Action (TRA)	2
					Social impact theory	2
					Self-regulation theory	2
					Theoretical Domains Framework (TDF)	2
					Protection motivation theory	1
					Social-ecological model	1
					Social support principles (Heaney and Israel)	1
					Social support theories	1
					Social norms theory	1
					Behavioral self-regulation model (C&S)	1
					Health education model	1
					EnRG framework	1
					Motivational interviewing	1
					Social identity theory	1
					Habit strength theory	1
					Dual process theory	1
					ANGELO model	1
					Perspectives on change model	1
					Knowledge-attitude-behavior model	1
					Cognitive Behavioral Therapy (CBT)	1
BCT Group 3						
5.2 Salience of consequences	26	.665	**Theory of planned behavior**	80	**Theory of planned behavior**	3
12.3 Avoidance/reducing exposure to cues for the behavior^a^	15	.651			Social cognitive theory	1
					TTM	1
6.3 Information about other’s approval	30	.599			HAPA	1
12.2 Restructuring the social environment	14	.498				
					PRIME	1
5.3 Information about social and environmental consequences	100	.475			TRA	1
					I-change	1
5.1 Information about health consequences	106	.460			TDF	1
13.2 Framing/reframing	43	.453				
					Implementation process theories	1
BCT Group 4						
8.1 Behavioral practice/rehearsal	104	.726	**Self-efficacy theory**	92	**Social cognitive theory**	40
6.1 Demonstration of the behavior	119	.621	**Social cognitive theory**	84	TPB	20
4.1 Instruction on how to perform the behavior	164	.563			TTM	11
					TRA	6
					**Self-efficacy theory**	6
					Health belief model	5
					TDF	5
					IMB skills model	4
					Social learning theory	4
					Self-determination	4
					CBT	4
					Self-regulation theory	3
					HAPA	2
					Control theory	2
					COM-B	2
					Operant learning theory	2
					I-change	2
					Health promotion model	2
					PRIME	1
					Protection motivation theory	1
					Health proportion model	1
					Social-ecological model	1
					Family-based theoretical framework	1
					Social inoculation theory	1
					Precaution adaptation process model	1
					Precaution adoption	1
					Organizational theory	1
					Self-management model	1
					Chronic disease self-management model	1
					Adapted physical activity model	1
					EnRG framework	1
					Motivational interviewing	1
					Planning	1
					Habit strength theory	1
					Dual process theory	1
					ANGELO model	1
					Knowledge-attitude-behavior model	1
					Elaboration likelihood model	1
					Frames model	1
					Adult learning theory	1
					Implementation process theories	1
					Habit theory	1
					Implementation intentions	1
					Implementation model	1
					Self-control theory	1
					Empowerment theory	1
BCT Group 5						
12.6 Body changes	14	.834			Social cognitive theory	4
10.9 Self-reward	15	.736			Self-efficacy theory	3
11.2 Reduce negative emotions	28	.509			Self-determination	2
					TTM	1
					PRIME	1
					Self-management model	1
					I-change	1
					Motivational interviewing	1
					CBT	1

Bold type indicates BCT Group–theory links supported by both expert consensus and literature synthesis.

^a^BCT 12.3 also loaded .582 on BCT Group 5.

Many of the intervention reports that explicitly reported the use of theory reported use of the same theories despite the set of BCTs used. Interventions that explicitly reported the Transtheoretical Model and Social Cognitive Theory were frequently reported for interventions that used a majority of the BCTs from any of the five BCT groups. Both the Theory of Planned Behavior and the Health Action Process Approach were frequently explicitly reported as underlying interventions that used BCTs similar to BCT Groups 1–4. Self-Determination Theory was frequently reported as underlying interventions that used BCTs similar to four groups—BCT Groups 1, 2, 4, and 5—and Self-Efficacy Theory for three groups—BCT Groups 2, 3, and 5. Based on these findings, we cannot draw conclusions about links between the five BCT groups and theory based on the authors’ explicit reports of theory use within individual interventions.

## Discussion

This study sought to identify co-occurring groups of BCTs in behavior change interventions and determine if behavior change theories underlying these groups could be identified. Five distinct groups of co-occurring BCTs were identified across a corpus of 277 intervention reports. This suggests that authors of interventions shared an explanatory model, regardless of whether it was stated explicitly, of which BCTs are perceived to work particularly well when combined. Next, experts reached consensus on a link between three groups of co-occurring BCTs and five theories. In the third phase, we compared the BCT group–theory links agreed by experts to the frequency with which authors reported using the same theories for published intervention descriptions using a set of BCTs that reflected a majority of the BCTs from a BCT group. For four out of the five expert consensus links between BCTs and the theories, several authors had also reported the same theories as a basis for their interventions.

BCT groupings were also reported as part of BCTTv1 based on expert grouping of BCTs with a similar mechanism of action [15]. It is possible to compare the groups of BCTs found in the current intervention reports with those produced by the BCTTv1 experts. In the current study, the first BCT group contained 13 BCTs, seven of which also belong to the BCTTv1 cluster “Goals and Planning” [15] linked by experts to two theories, “Health Action Process Approach” and “Self-Regulation Theory,” which involve goals and planning. Although only three authors explicitly made a similar link, most theories that authors did mention were broadly social cognition theories that include the identification of goals or intentions. BCT Group 3 included seven BCTs directed at changing behavioral and normative beliefs, three of which belong to the BCTTv1 grouping “Natural Consequences” and two of which belong to the BCTTv1 grouping “Antecedents”; this group was linked by both experts and intervention report authors to the “Theory of Planned Behavior.” BCT Group 4 contained BCTs that prompt instruction, demonstration, and practice of a desired behavior and all emphasize modeling, skill practice, and development. The experts and several intervention reports linked this group of BCTs to “Social Cognitive Theory” and “Self Efficacy Theory, two theories that have much in common. BCT Group 4 was also linked to the “Theory of Planned Behavior” by a sizable number of intervention authors, which could reflect a focus on specific overlapping theoretical constructs like perceived behavioral control (Theory of Planned Behavior) and self-efficacy (Social Cognitive Theory and Self-Efficacy Theory).

We found that experts can agree on theories underlying a group of BCTs and the theories experts identified were explicitly reported within intervention reports using a similar group of BCTs. However, two BCT groups from the factor analysis did not fit any of the vast number of possible theories, suggesting an underlying intuitive rather than formal model of the process of change. Furthermore, the observed grouping of BCTs did not reliably indicate the authors’ stated theorizing within the intervention reports. This could be due to factors such as suboptimal use of theory in intervention design, including post hoc labeling of intervention technique groupings with theory, repeated testing of ideas rooted in common sense rather than theory, or incomplete intervention content reporting obscuring the association.

It has not been possible to draw strong conclusions in the current study, but it does point to issues in this field. As argued earlier, theory is increasingly being recommended for use in intervention development and evaluation. In the current study, 64% of authors stated the theory (or theories) on which their intervention was based—suggesting that intervention authors are indeed referencing theories as they develop interventions. However, the results cannot speak to the precision or accuracy with which theoretical principles and intervention techniques are linked. In future, researchers might benefit from adopting tools such as the Theory Coding Scheme [30] to evaluate and report their use of theory. Second, even experienced behavior change intervention experts did not feel “expert” in using this range of theories, possibly contributing to some of the lack of certainty about links with BCT groups. The plethora of theories available in the literature may also be a source of confusion for intervention developers. The discussion among experts during the current study echoed a concern about the abundance of theories, which emphasizes the potential for confusion among less experienced intervention developers. Some method of reducing the number of theories, rather than expanding this number without strong reasoning, might be helpful in developing a coherent approach to theory use for intervention development more broadly. One such approach is the application of an ontological modeling system to integrate a large corpus of theories of behavior change [31].

A previous attempt to aggregate and specify theories used in behavior change interventions identified 83 theories with over 1,700 theoretical constructs; it is clear that this may leave intervention developers with an unmanageable range of possible mechanisms of change to target in interventions [25]. Earlier studies in the current program of research examined the links between BCTs and mechanisms of action [12, 22, 23, 32] in order to assist intervention developers in their choice of BCTs and found support for 92 possible links between them [32]. The current study takes this one step further, moving to formal theories and from individual BCTs to groups of co-occurring BCTs.

It has been argued that the lack of an overarching theoretical framework prevents replicability and, therefore, cumulative science on behavioral topics [3]. With over 70 overlapping theories referenced in 277 published intervention reports, we appear some way distant from such a framework [19]. While interventions frequently use similar content, and similar theories are frequently reported as informing interventions, there does not appear to be coherence between the techniques frequently used as a group and the theories informing these groups of techniques. It is also not clear whether intervention reports using the same theory apply similar BCTs. We have examined whether co-occurring BCTs link to a recognizable theory; we have not examined whether authors proposing the same theory have used similar BCTs. Earlier stages of this program of research identified links between BCTs and theoretical constructs [12, 22, 32]; identifying theoretical constructs within theories provides a basis for proposing how BCTs might be combined in interventions based on a particular theory [31]. The potential to develop an overarching theoretical framework based on existing theories has increased with an ontology-based modeling system developed for representing behavior change theories, with the successful representation of 77 theories both in diagrams and a computer-readable format as a searchable database [31].

## Limitations

The BCT groups evaluated in this study were restricted to those BCTs identified as co-occurring within 277 behavior change interventions, these BCT groups could represent only a subset of possible groups of BCTs that might co-occur across all behavior change interventions. Further, the BCT groups may be more indicative of recent and/or “popular” trends in behavioral theory research given the representation of more recent papers in the current data set. The results of the factor analysis suggested possible alternative numbers of factors with an acceptable model fit, and we chose the factor solution with the fewest number of cross-loading BCTs. Selecting a parsimonious factor solution may have had an impact on the findings and a different factor solution may have yielded different results. However, given that the one BCT that cross-loaded onto another factor was also one of the two BCTs, which more than one expert agreed to drop from a BCT group, (BCT 12.3), the extent to which a different factor solution would have produced better results seems limited. Furthermore, the examination of alternative factor solutions with acceptable model fit suggested solutions that made less conceptual sense; this could be related to the overlap across behavior change theories.

Other factors that may limit the findings concern the conduct of the consensus exercise. The 25 experts who rated links between the BCT groups and theories predominantly work in the health psychology field and, possibly, a larger number of experts and/or different recruitment strategies would have yielded different experts and ultimately different findings. However, these experts were able to reach a satisfactory level of consensus. Experts were given a three-point rating scale to assess the link between BCT groups and theory, with an “Uncertain/Don”t Know” midpoint. It is possible that providing separate response options for “uncertain” and “do not know” might have permitted greater coherence among the experts. Furthermore, experts may have linked BCT groups to theory using a “best fit” approach, which may be inexact compared to linking BCT groups at the construct level. However, this limitation should be softened by experts’ option to add or remove BCTs from the BCT group to improve the link with a specified theory. Nevertheless, given the experts’ lack of confidence in their ratings and the limitations of exploratory factor analyses, further investigation of the current research questions is warranted.

## Future Directions

Additional research should examine these and other groups of BCTs that co-occur across a broader range of behavior change interventions and whether the groups of BCTs that co-occur across interventions differ by intervention mode of delivery (e.g., in-person vs. digital interventions) or by behavior domain (e.g., smoking vs. diet). The results of this study could be compared to previous work by examining the individual BCTs within a BCT group, which were previously linked individually to mechanisms of action [22, 33], to determine the extent to which the linked mechanisms of action are theoretical constructs present within the behavior change theory that experts and the literature linked to a given BCT group. The factor analyses used to group BCTs were exploratory and further research is needed to ascertain whether these or other groupings of BCTs are commonly found in the literature. The experts reported low confidence in their use of theory and further evidence is needed about the links between BCT groups and theory, perhaps restricting the range of theories and ensuring confident expertise in at least some of the more commonly cited theories. Future research might also consider replicating these findings within different intervention reports and/or with different approaches to examining whether there are converging results. Further synthesis of existing behavior change interventions will produce incremental evidence to advance the science of behavior change, with an eye toward a more encompassing theoretical change framework that maps to BCTs.

## Conclusions

The findings from this study indicate that BCTs are reliably grouped in behavior change interventions and experts can reach a systematically drawn consensus about the correspondence between groups of BCTs and behavior change theories. The findings demonstrate a potentially shared theorizing about how BCTs may work together in interventions and how this shared theorizing may be driven by behavior change theory, whether this theory was agreed by experts, stated by multiple interventions, and, in some cases, both. This information can inform intervention development and synthesis by improving systematic thinking about interventions, including a means to evaluate whether the use of specific BCT groups is associated with significant outcomes. Improving clarity on how behavior change theories inform the authors’ selection of groups of BCTs to use in an intervention has the potential to provide a framework for specifying how interventions have their effects and, in turn, the evaluation of those predictions. Additionally, these results can be used in conjunction with results from this program of research to examine how BCTs frequently used together in an intervention have been individually linked to mechanisms of action. The evidence from this type of work will inform efforts to advance intervention design and behavioral theory.

## Supplementary Material

kaaa078_suppl_Supplementary_Material
