# Supplementary material for: Do Combinations of Behavior Change Techniques That Occur Frequently in Interventions Reflect Underlying Theory?
Source: Ann Behav Med. 2020 Sep 22;54(11):827–42. doi: 10.1093/abm/kaaa078 (PMC7646153; doi:10.1093/abm/kaaa078)
Supplement: kaaa078_suppl_Supplementary_Material [file kaaa078_suppl_supplementary_material.pdf]

Electronic Supplementary Materials

Supplementary File 1. *Scree plot of eigenvalues from exploratory factor analysis*

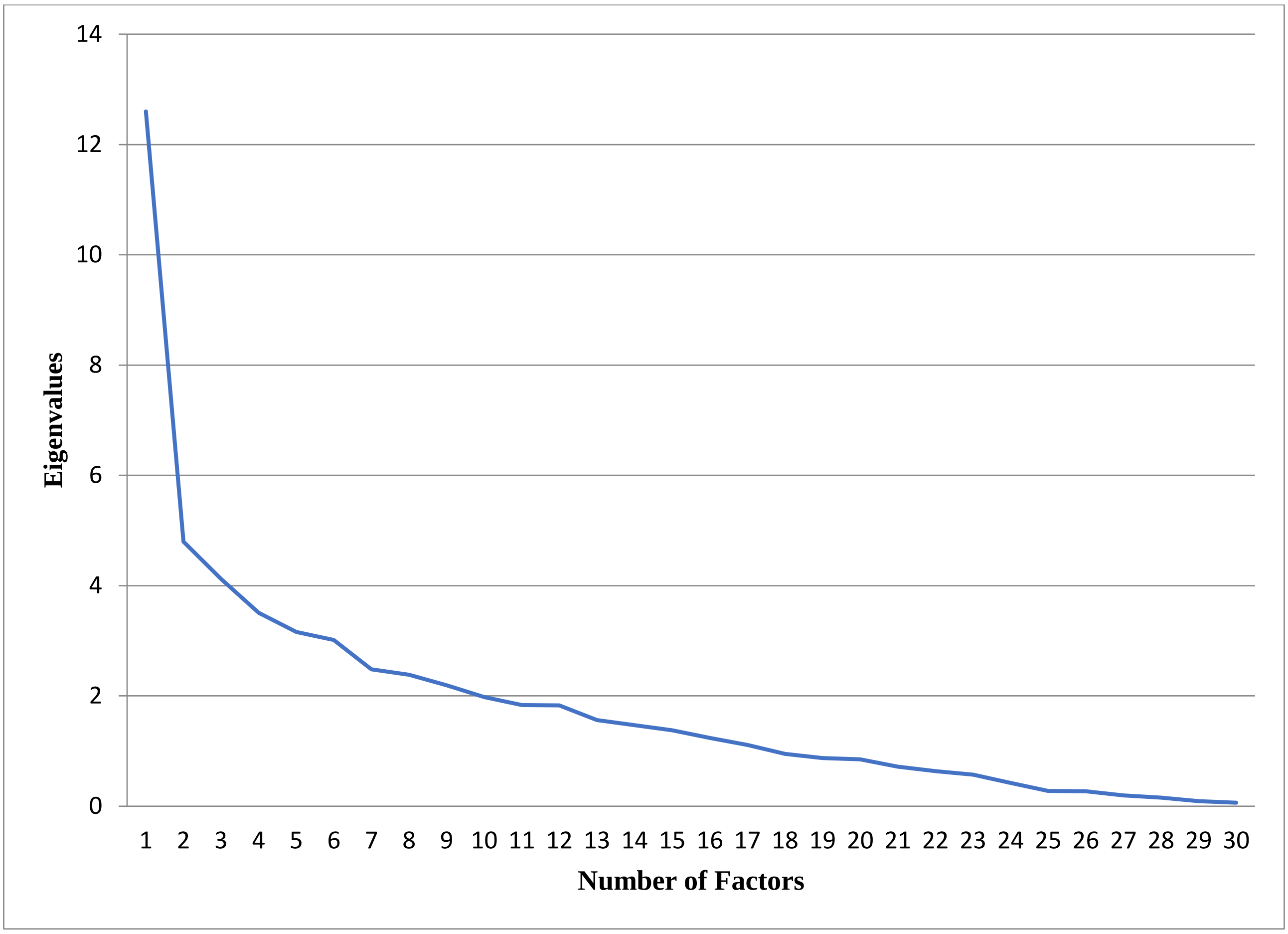

Supplementary File 2. *BCT labels and definitions presented by BCT Group*

BCT Group	Factor Loading	BCTTV1 Number & Label	BCT Definition
BCT Group 1	823	1.5 Review Behaviour Goals	Review behaviour goal(s) jointly with the person and consider modifying goal(s) or behaviour change strategy in light of achievement. This may lead to re-setting the same goal, a small change in that goal or setting a new goal instead of (or in addition to) the first, or no change.
	.783	1.4 Action Planning	Prompt detailed planning of performance of the behaviour (must include at least one of context, frequency, duration and intensity). Context may be environmental (physical or social) or internal (physical, emotional or cognitive).
	. 729	15.3 Focus on Past Success	Advise to think about or list previous successes in performing the behaviour (or parts of it).
	.717	1.1 Goal Setting (Behaviour)	Set or agree a goal defined in terms of the behaviour to be achieved.
	.712	15.4 Self-Talk	Prompt positive self-talk (aloud or silently) before and during the behaviour.
	.705	3.3 Social Support (Emotional)	Advise on, arrange, or provide emotional social support (*e.g. from friends, relatives, colleagues, 'buddies' or staff*) for performance of the behaviour.
	.624	1.2 Problem Solving	Analyse, or prompt the person to analyse, factors influencing the behaviour and generate or select strategies that include overcoming barriers and/or increasing facilitators
	.613	2.3 Self-Monitoring of Behaviour	Establish a method for the person to monitor and record their behaviour(s) as part of a behaviour change strategy.
	.570	8.7 Graded Tasks	Set easy-to-perform tasks, making them increasingly difficult, but achievable, until behaviour is performed.
	.560	1.3 Goal Setting (Outcome)	Set or agree a goal defined in terms of a positive outcome of wanted behaviour.
	.523	1.6 Discrepancy between Current Behaviour and Goal	Draw attention to discrepancies between a person's current behaviour (in terms of the *form, frequency, duration,* or *intensity* of that behaviour) and the person's previously set outcome goals, behavioural goals or action plans (goes beyond self-monitoring of behaviour).
	.503	2.4 Self-Monitoring of Outcomes of Behaviour	Establish a method for the person to monitor and record the outcome(s) of their behaviour as part of a behaviour change strategy.
	.489	1.8 Behavioural Contract	Create a written specification of the behaviour to be performed, agreed by the person, and witnessed by another.
BCT Group 2	.807	2.7 Feedback on Outcomes of Behaviour	Monitor and provide feedback on the outcome of performance of the behaviour.
	.740	2.2 Feedback on Behaviour	Monitor and provide informative or evaluative feedback on performance of the behaviour (e.g. *form, frequency, duration, intensity*).

	.626	6.2 Social Comparison	Draw attention to others' performance to allow comparison with the person's own performance
BCT Group 3	.665	5.2 Salience of Consequences	Use methods specifically designed to emphasise the consequences of performing the behaviour with the aim of making them more memorable (goes beyond informing about consequences).
	.651	12.3 Avoidance/Reducing Exposure to Cues for the Behaviour	Advise on how to avoid exposure to specific social and contextual/physical cues for the behaviour, including changing daily or weekly routines.
	.599	6.3 Information about Others’ Approval	Provide information about what other people think about the behaviour. The information clarifies whether others will like, approve or disapprove of what the person is doing or will do. 3
	.498	12.2 Restructuring the Social Environment	Change, or advise to change the **social** environment in order to facilitate performance of the wanted behaviour or create barriers to the unwanted behaviour (other than prompts/cues, rewards and punishments).
	.475	5.3 Information about Social and Environmental Consequences	Provide information (*e.g. written*, *verbal*, *visual*) about social and environmental consequences of performing the behaviour.
	.460	5.1 Information about health consequences	Provide information (*e.g. written*, *verbal*, *visual*) about health consequences of performing the behaviour.
	.453	13.2 Framing/Reframing	Suggest the deliberate adoption of a perspective or new perspective on behaviour (*e.g. its purpose*) in order to change cognitions or emotions about performing the behaviour (includes 'Cognitive structuring').
BCT Group 4	.726	8.1 Behavioural Practice / Rehearsal	Prompt practice or rehearsal of the performance of the behaviour one or more times in a context or at a time when the performance may not be necessary, in order to increase habit and skill.
	.621	6.1 Demonstration of the Behaviour	Provide an observable sample of the performance of the behaviour, directly in person or indirectly (*e.g. via film, pictures, for the person to aspire to or imitate*), includes 'Modelling'.
	.563	4.1 Instruction on How to Perform the Behaviour	Advise or agree on how to perform the behaviour (includes 'Skills training').
BCT Group 5	.834	12.6 Body Changes	Alter body structure, functioning or support **directly** to facilitate behaviour change.
	.736	10.9 Self-Reward	Prompt self-praise or self-reward if and only if there **has been** effort and/or progress in performing the behaviour.
	.509	11.2 Reduce Negative Emotions	Advise on ways of reducing negative emotions to facilitate performance of the behaviour (includes 'Stress Management').

Note: Labels and definitions from: Michie S, Richardson M, Johnston M, et al.: The Behavior Change Technique Taxonomy (v1) of 93 Hierarchically Clustered Techniques: Building an International Consensus for the Reporting of Behavior Change Interventions. *Ann Behav Med.* 2013, *46:*81-95.

Supplementary File 3. *Content of the Guidelines for Rounds 1-4 of the Consensus Exercise*

Overview

Background & Aim

The Theories and Techniques of Behaviour Change Project (www.ucl.ac.uk/behaviour-change-techniques) aims to identify hypothesised links between behaviour change techniques (BCTs) and (i) the mechanisms of action through which they influence behaviour and (ii) behavioural theories. The final phase of this research aims to examine whether groups of co-occurring BCTs can be linked to specific theories.

Participants

You are one of 25 experts from 10 countries, selected because you have a high level of expertise in theories and BCTs and provided valuable input in our previous consensus exercise.

Overview of Study

The consensus exercise is a modified Nominal Group Technique, involving 4 rounds. In Round 1, you will be directed to an online questionnaire and asked to list all possible theories that might underlie a particular BCT group. You will be asked to do this for 5 BCT groups (see Appendix A). In Round 2, you will be presented with the same 5 BCT groups, and asked to consider the extent to which each BCT group is linked to a particular theory; the theories you are asked to consider will be those identified by experts in Round 1. In Round 3, you will be invited to participate in an online, asynchronous (i.e. each member can participate at a time of their choosing), anonymous discussion, in which you will be encouraged to exchange views with other experts. Finally, in Round 4, you will be asked to provide final ratings on links between BCT groups and theories (which may or may not be the same as your Round 2 ratings). At this point, you will also have the opportunity to add and/or remove BCTs from each BCT group for each theory.

More detail on Rounds 1 and 2 can be found below; Guidelines for Rounds 3 and 4 will be sent out separately.

Round 1 Guidelines

Information Provided

In Appendix A, you will find 5 groups of co-occurring BCTs (i.e. BCTs that were found to appear together frequently in interventions, identified through an exploratory factor analysis). These BCT groups will be presented to you on screen during the consensus exercise. In addition, you will receive a complementary online book containing a description of 83 theories (‘ABC of Behaviour Change Theories’), which you may draw on during the task. Log-in details to access this online book will be sent to you by email.

Question

You will be presented with 5 BCT groups, one at a time, and asked to list all theories that you consider to be associated with each group, in the following question:

> **An intervention was developed that includes the BCTs listed above. Which behavioural theory or theories do you think could have guided the development of this intervention?**

Response Options

To respond, you will be provided with an open text box in which you can list one or more theories, drawing on the ‘ABC of Behaviour Change Theories’ and/or your own knowledge and expertise (see Appendix B for a preview of the Round 1 questionnaire).

Round 2 Guidelines

Information Provided

You will have access to the same information as above (i.e. the 5 BCT groups and the online book, 'ABC of Behaviour Change Theories').

Question

You will be presented with 5 BCT groups, one at a time, and asked to respond to the following question for all theories listed by experts in Round 1:

An intervention was developed that includes the BCTs listed above. How confident are you that this group of BCTs is linked to [Theory X]?

Response Options

a) 'Very confident': Very confident that this group of BCTs is linked to [Theory X]
b) 'Uncertain/Don't Know': Uncertain about whether or not this group of BCTs is linked to [Theory X]
c) 'Not at all confident': Not at all confident about whether or not this group of BCTs is linked to [Theory X]

When providing your ratings, please remember:

(1) In order to ensure consistency across experts, and with the aim of developing a shared understanding of BCT-mechanism of action links, it is very important that you base your answers on the **definitions**, rather than labels, of the BCTs.
   - Definitions for all BCTs will appear on the screen during the consensus exercise, and these can also be found in Appendix A. Please read and re-read these definitions before beginning the task. Definitions can also be accessed via www.bcts.23.co.uk, or through the BCTTv1 smartphone app, which can be downloaded for free via the Apple App and Google Play stores.

(2) In answering the questions, you may draw on your own knowledge and expertise and/or the theories in the 'ABC of Behaviour Change Theories'.

Round 3 Guidelines

Overview:
Thank you for completing the first two rounds of this study. In Round 3, we are asking for your input on links between groups of co-occurring BCTs and theories where there remains uncertainty and/or disagreement. The purpose of this round is to discuss these uncertainties and disagreements to help guide your final round ratings. By providing your input in this round, you will add to our shared understanding of the links between BCT groups and theories, and help to build consensus. This is your opportunity to share your thoughts with other behaviour change experts about the task.

Information Provided:
In Appendix A, you will find confidential data from Round 2 of this consensus exercise, including a summary of all experts' responses, and a reminder of your own responses. You will be able to see the group data on screen as part of the Round 3 discussion. However, we recommend that you read through Appendix A in full before Round 3 begins, in order to give you a chance to reflect on the data to inform your contribution to the discussion.

In Appendix B, you will find step-by-step instructions for how to use 'Loomio', the online discussion forum on which Round 3 will be hosted.

What the task involves:

- We are asking experts to contribute to an online discussion forum, in which you will have the opportunity to share your views about BCT group-theory links rated in Round 2, and to comment on other experts' views.
- The online discussion will involve the group of 25 experts who are participating in this study. Your contributions will be anonymous (i.e. you will use an assigned Expert ID and not your name).
- We estimate that your participation in Round 3 should take approximately 1.5 hours in all. While you may log in and participate at any time(s) of your choosing, we would recommend that you log in at least three times over the Round 3 period. This is to ensure that you have had a chance to contribute fully to the discussion.
- A moderator from the research team will periodically summarise the discussion and raise issues for further consideration.

How to participate:
Step-by-step instructions for participating in the online discussion can be found in Appendix B. In summary:

- You will be sent a link to the discussion via an email from the research team. When you click on this link, a website called 'Loomio' will open and you will be prompted to register an account or log in. You can log in using the details you previously registered (i.e. during the last expert consensus study). If, for any reason, you need to register a new account (e.g. if you no longer have a Loomio account), it is very important that you do this using your Expert ID and not your name.

- There will be a number of discussion groups on which you can comment, each specific to a BCT group-theory link for which there was high uncertainty or disagreement in Round 2 (see Appendix A). There will be other discussion groups in which you can contribute thoughts about the task more generally and/or about other BCT group-theory links.

- It may be helpful to focus on the ratings of BCT group-theory links for which (i) you remain very uncertain, and/or (ii) your responses differed from those of the other experts. However, we would also value your thinking on other aspects of the task.

Round 4 Guidelines

Overview

Thank you for completing the first three rounds of this study. In Round 4, we are asking you to provide your final ratings for links between groups of behaviour change techniques (BCTs) and behavioural theories. You will be directed to an online questionnaire and asked to consider whether a group of BCTs is linked to a particular theory. In addition, for each BCT group, you will be asked to indicate the theory or theories (up to three) you are *most* confident are linked. For these theories, you will also have the opportunity to consider whether there are BCTs that should be added to and/or removed from the BCT group.

Information provided:

During the final round, you will see data on screen from Round 2 (i.e. 'In Round 2, X% of experts were very confident that this BCT group is linked to this theory'). For the links that were discussed in Round 3, there will be hyperlinks to the relevant discussion page on Loomio (note that you will need to be signed in to view this content). You will not be asked to discuss links for which there was consensus in Round 2.

Definitions for the BCTs in each BCT group will be presented on screen during the consensus exercise (these are also available in Appendix A). BCT definitions can also be accessed via www.bcts.23.co.uk, and through the BCTTv1 smartphone app, which can be downloaded for free via the Apple App and Google Play stores.

To view theory descriptions, you may draw on the ABC of Behaviour Change Theories book, as in the previous rounds.

What the task involves:

1. You will be presented with 5 BCT groups, one at a time, and asked to respond to the following question (for all theories where consensus was not reached in Round 2):

> **An intervention was developed that includes the BCTs listed above.**
> **How confident are you that this group of BCTs is linked to [Theory X]?**

Response Format

- In this round, the response options for this question will be:

> a) **'Confident that they are linked':** Confident that this group of BCTs is linked to [Theory X].
> *Note*: You may use this option if you believe *most* (i.e. not necessarily all) of the BCTs in a group are linked to this theory.
> b) **'Uncertain/Don't Know':** Uncertain about whether or not this group of BCTs is linked to [Theory X].
> c) **'Confident that they are not linked':** Confident that this group of BCTs is not linked to [Theory X].

2. Following completion of the above, you will be asked to identify the theory or theories that you are most confident are linked to each BCT group.
   - For each BCT group, you will be presented with the theories you rated as 'confident that there is a link' and asked to select those you are most confident about.
   - You may base your judgements on the frequency and/or strength of links between the BCTs within a group and the constructs of the theory.

Response Format

- The response format for this question will be a click-and-drag format. You will be asked to select a theory and ‘drag’ it into the ‘most confident’ box. You will be able to do this for up to three theories. If you select more than one theory, you will be able to re-order theories within the box.

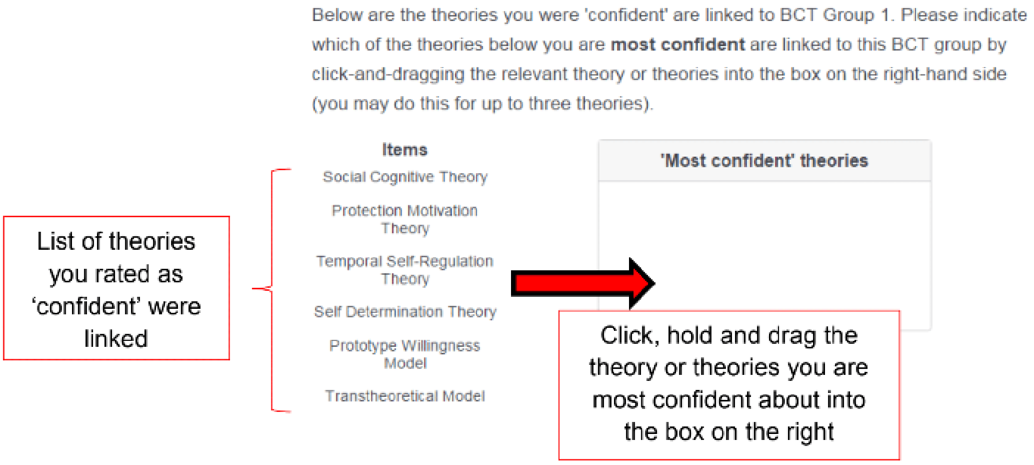

3. Finally, for each of the theories you identified in the previous step, you will be asked to indicate whether or not there are BCTs you think should be added to, or removed from, the BCT group. You will be provided with an open text box to indicate which (if any) BCTs you would add and/or remove.

Supplementary File 4. *Names of theories linked to Behaviour Change Technique (BCT) Groups by more than two experts in the expert consensus exercise*

	BCT Group 1	BCT Group 2	BCT Group 3	BCT Group 4	BCT Group 5
Theories Linked (*n*)	36	45	68	25	20
	Action Theory Model of Consumption	Action Theory Model of Consumption	Action Theory Model of Consumption		
					Affective Events Theory
			AIDS Risk Reduction Model		
			Behavioral Ecological Model of Adolescent AIDS Prevention		
	CEOS Theory	CEOS Theory	CEOS Theory		CEOS Theory
		Change Theory	Change Theory		
			Classical Conditioning Theory	Classical Conditioning Theory	Classical Conditioning Theory
	COM-B Model	COM-B Model	COM-B Model	COM-B Model	COM-B Model
			Consumption and Social Practice Theory		
			Containment Theory		
	Control Theory	Control Theory	Control Theory		
			Differential Association Theory		
	Diffusion of Innovations Theory	Diffusion of Innovations Theory	Diffusion of Innovations Theory	Diffusion of Innovations Theory	
			Ecological Model for Preventing Type 2 Diabetes in Minority Youth		
			Extended Information Processing Model		
		Extended Parallel Process Model	Extended Parallel Process Model		Extended Parallel Process Model
	Feedback Intervention Theory	Feedback Intervention Theory			
	Focus Theory of Normative Conduct	Focus Theory of Normative Conduct	Focus Theory of Normative Conduct		
	General Theory of Crime		General Theory of Crime		
		General Theory of Deviant Behavior	General Theory of Deviant Behavior		
	Goal Directed Theory	Goal Directed Theory			
			Goal Framing Theory		
	Goal Setting Theory	Goal Setting Theory		Goal Setting Theory	Goal Setting Theory
	Health Action Process Approach	Health Action Process Approach	Health Action Process Approach	Health Action Process Approach	Health Action Process Approach

Health Behavior Goal Model	Health Behavior Goal Model	Health Behavior Goal Model		
		Health Behavior Internalization Model		
Health Belief Model	Health Belief Model	Health Belief Model	Health Belief Model	
Health Promotion Model	Health Promotion Model	Health Promotion Model	Health Promotion Model	
I-Change Model	I-Change Model	I-Change Model	I-Change Model	
Information-Motivation-Behavioural Skills Model		Information-Motivation-Behavioural Skills Model	Information-Motivation-Behavioural Skills Model	
		Information-Motivation-Behavioural Skills Model of Adherence		
Integrated Model of Behavioural Prediction		Integrated Model of Behavioural Prediction	Integrated Model of Behavioural Prediction	
		Integrated Theoretical Model for Alcohol and Other Drug Abuse Prevention	Integrated Theoretical Model for Alcohol and Other Drug Abuse Prevention	
		Integrated Theory of Drinking Behavior		
Integrated Theory of Health Behaviour Change (Ryan)	Integrated Theory of Health Behaviour Change (Ryan)	Integrated Theory of Health Behaviour Change (Ryan)	Integrated Theory of Health Behaviour Change (Ryan)	Integrated Theory of Health Behaviour Change (Ryan)
	Integrative Model of Factors Influencing Smoking and Attitude and Health Behavior Change	Integrative Model of Factors Influencing Smoking and Attitude and Health Behavior Change		
	Integrative Model of Factors Influencing Smoking Behaviors	Integrative Model of Factors Influencing Smoking Behaviors		Integrative Model of Factors Influencing Smoking Behaviors
Integrative Model of Health Attitude and Behaviour Change (Flay)		Integrative Model of Health Attitude and Behaviour Change (Flay)		Integrative Model of Health Attitude and Behaviour Change (Flay)
		Model of Pro-Environmental Behavior		
	Motivation-Opportunities-Abilities Model	Motivation-Opportunities-Abilities Model	Motivation-Opportunities-Abilities Model	
	Norm Activation Theory			
	Operant Learning Theory	Operant Learning Theory	Operant Learning Theory	Operant Learning Theory
		Precaution Adoption Process Model		

PRIME Theory	PRIME Theory	PRIME Theory		PRIME Theory
	Problem Behavior Theory	Problem Behavior Theory	Problem Behavior Theory	
		Prospect Theory		
Protection Motivation Theory	Protection Motivation Theory	Protection Motivation Theory		Protection Motivation Theory
Prototype Willingness Model	Prototype Willingness Model	Prototype Willingness Model		
Reflective Impulsive Model		Reflective Impulsive Model		
Regulatory Fit Theory				
Relapse Prevention Model	Relapse Prevention Model	Relapse Prevention Model	Relapse Prevention Model	Relapse Prevention Model
				Risk as Feelings Theory
Self Determination Theory	Self Determination Theory	Self Determination Theory	Self Determination Theory	Self Determination Theory
Self-Efficacy Theory	Self-Efficacy Theory	Self-Efficacy Theory	Self-Efficacy Theory	Self-Efficacy Theory
Self-Regulation Theory	Self-Regulation Theory	Self-Regulation Theory		
		Six Staged Model of Communication Effects	Six Staged Model of Communication Effects	
	Social Action Theory (Ewart)	Social Action Theory (Ewart)		
	Social Action Theory (Weber)	Social Action Theory (Weber)		
	Social Change Theory	Social Change Theory		
Social Cognitive Theory	Social Cognitive Theory	Social Cognitive Theory	Social Cognitive Theory	Social Cognitive Theory
		Social Consensus Model of Health Education		
		Social Development Model		
Social Ecological Model of Behavior Change	Social Ecological Model of Behavior Change			
	Social Identity Theory	Social Identity Theory		
	Social Influence Model of Consumer Participation	Social Influence Model of Consumer Participation		
	Social Learning Theory	Social Learning Theory	Social Learning Theory	
	Social Norms Theory	Social Norms Theory		
Systems Model of Health Behaviour Change	Systems Model of Health Behaviour Change	Systems Model of Health Behaviour Change	Systems Model of Health Behaviour Change	
Temporal Self-Regulation Theory		Temporal Self-Regulation Theory		
Theory of Interpersonal Behaviour	Theory of Interpersonal Behaviour	Theory of Interpersonal Behaviour	Theory of Interpersonal Behaviour	

		Theory of Normative Social Behavior	Theory of Normative Social Behavior		
	Theory of Planned Behavior	Theory of Planned Behavior	Theory of Planned Behavior	Theory of Planned Behavior	Theory of Planned Behavior
			Theory of Reasoned Action		
		Theory of Triadic Influence	Theory of Triadic Influence		
	Transcontextual Model of Motivation		Transcontextual Model of Motivation		
	Transtheoretical Model of Behavior Change		Transtheoretical Model of Behavior Change	Transtheoretical Model of Behavior Change	Transtheoretical Model of Behavior Change

Supplementary File 5. *Links between theories and BCT groups agreed not to be present by ≥80% experts*

BCT Group	Behaviour Change Theory	Experts 'not at all confident' in the link *(%)*
BCT Group 1	Health Belief Model	80
	Focus Theory of Normative Feedback	88
	Diffusion of Innovations Theory	87.5
	Prototype Willingness Model	83.33
BCT Group 2	Protection Motivation Theory	83.33
BCT Group 3	Control Theory	87.5
	Diffusion of Innovations Theory	83.33
	Operant Learning Theory	83.33
	Self-Efficacy Theory	83.33
	Social Identity Theory	87.5
	Temporal Self-Regulation Theory	83.33
BCT Group 4	Classical Conditioning Theory	83.33
	Diffusion of Innovations Theory	80
	Health Belief Model	80
BCT Group 5	Self Determination Theory	87.5
	Goal Setting Theory	83.33
	Theory of Planned Behaviour	83.33
